# ﻿Assessment of quadrivalent characteristics influencing chromosome segregation by analyzing human preimplantation embryos from reciprocal translocation carriers

**DOI:** 10.3897/compcytogen.18.115070

**Published:** 2024-01-22

**Authors:** Ziravard N. Tonyan, Irina L. Puppo, Alsu F. Saifitdinova, Tatyana V. Vavilova, Andrey S. Glotov

**Affiliations:** 1 D. O. Ott Research Institute of Obstetrics, Gynaecology and Reproductology, 3 Mendeleevskaya Line, 199034, Saint Petersburg, Russia Almazov National Medical Research Centre Saint Petersburg Russia; 2 Almazov National Medical Research Centre, 2 Akkuratova Street, 197341, Saint Petersburg, Russia D. O. Ott Research Institute of Obstetrics, Gynaecology and Reproductology Saint Petersburg Russia; 3 International Centre for Reproductive Medicine, 53/1 Komendantskij prospect, 197350, Saint Petersburg, Russia International Centre for Reproductive Medicine Saint Petersburg Russia; 4 Department of Human and Animal Anatomy and Physiology, Herzen State Pedagogical University of Russia, 48 Moyka River Embankment, 191186, Saint Petersburg, Russia Herzen State Pedagogical University of Russia Saint Petersburg Russia

**Keywords:** Chromosome segregation, PGT-SR, quadrivalent asymmetry, reciprocal translocation, segregation, terminal breakpoints

## Abstract

Patterns of meiotic chromosome segregation were analyzed in cleavage stage and blastocyst stage human embryos from couples with autosomal reciprocal translocations (ART). The influence of quadrivalent asymmetry degree, the presence of terminal breakpoints, and the involvement of acrocentric chromosomes in the rearrangement were analyzed to evaluate their contribution to the formation of non-viable embryos with significant chromosomal imbalance due to pathological segregation patterns and to assess the selection of human embryos by the blastocyst stage. A selection of viable embryos resulting from alternate and adjacent-1 segregation and a significant reduction in the detection frequency of the 3 : 1 segregation pattern were observed in human embryos at the blastocyst stage. The presence of terminal breakpoints increased the frequency of 3 : 1 segregation and was also associated with better survival of human embryos resulting from adjacent-1 mode, reflecting the process of natural selection of viable embryos to the blastocyst stage. The demonstrated patterns of chromosome segregation and inheritance of a balanced karyotype in humans will contribute to optimizing the prediction of the outcomes of in vitro fertilization programs and assessing the risks of the formation of unbalanced embryos for ART carriers.

## ﻿Introduction

Autosomal reciprocal translocations (ART) are balanced structural rearrangements resulting from the interchange among two or more non-homologous chromosomes with an exchange of the fragments (Gardner and Amor 2018). ARTs can contribute to karyotype evolution by altering the structure and organization of chromosomes in germ cells and early embryonic cells (Imai et al. 1986). It is estimated that the overall frequency of reciprocal translocations in *Homosapiens* Linnaeus, 1758 is 1 : 500 (Ogilvie et al. 2005). Carriers of balanced ART have a normal phenotype, but the risk of producing genetically unbalanced gametes is increased due to malsegregation of chromosomes during meiosis. As a result, ART can lead to recurrent miscarriage, infertility, or the birth of a child with multiple congenital malformations caused by a chromosomal abnormality (De Braekeleer and Dao 1990).

Derivative chromosomes and their normal homologous form a special structure called quadrivalent in the first meiotic division in ART carriers. Quadrivalent chromosomes segregate in one of the following modes: 2 : 2, 3 : 1, or 4 : 0. Alternate segregation is the type of 2:2 segregation, which is characterized by the inheritance by each of the daughter cells of two normal or two derivative chromosomes. It is the only segregation pattern that does not result in the formation of unbalanced gametes. Adjacent-1 and adjacent-2 are two more types of 2 : 2 segregation, leading to the formation of a zygote with partial trisomy for one of the translocated segments (TS) and monosomy for the other TS or with partial trisomy for one of the centric segments (CS) and monosomy for the other CS, respectively. The 3 : 1 mode results in trisomy or monosomy for one of the rearranged or normal chromosomes involved in translocation. The 4 : 0 segregation pattern leads to complete trisomy or complete monosomy for both chromosomes involved in the rearrangement (Gardner and Amor 2018).

Different approaches can be used to analyze chromosome segregation in ART carriers and the factors influencing it. The analysis of the genetic content of polar bodies could provide valuable insights into the nature of meiotic segregation (Kuliev and Verlinsky 2004; Magli et al. 2011). However, during early embryonic development, the first polar bodies are typically eliminated (Fabian et al. 2012), which complicates their use for conducting systematic research. Since there is no activation of the embryo genome before the cleavage stage of human embryonic development, which typically occurs on the third day after fertilization (Dobson et al. 2004), significant selection of genetically unbalanced embryos does not occur until the third day of development. This provides researchers with a unique opportunity to study the segregation of rearranged chromosomes in the gametogenesis of ART carriers by analyzing cleavage stage human embryos. However, it should be noted that according to recent data, activation of the embryonic genome in humans initiates at the single-cell stage (Asami et al. 2022). As the embryo’s genome becomes activated, it starts to transcribe and translate its own genetic material, leading to the production of proteins and molecules necessary for further development and differentiation. A key marker of genome activation of the human embryo is the compaction of genetic material, which is crucial for the embryo’s growth and the formation of the blastocyst (Hur et al. 2023). Modern techniques in assisted reproductive technology have advanced the ability to culture and grow blastocysts in vitro (Sills and Palermo 2010). However, not all unbalanced embryos reach the blastocyst stage due to the natural selection of genetically imbalanced embryos by the fifth or sixth day of development (Beyer and Willats 2017).

Studies analyzing the contribution of various factors to chromosome segregation in human embryos at different stages of development demonstrated the influence of the quadrivalent asymmetry degree, the presence of terminal breakpoints, and the participation of the acrocentric chromosome in the rearrangement.

For instance, a higher frequency of the formation of genetically normal/balanced embryos was demonstrated in the absence of terminal breakpoints when analyzing cleavage stage embryos (Ye et al. 2012). The same trend was shown in blastocysts, but the presence of terminal breakpoints also predisposed to adjacent-1 segregation (Xie et al. 2022). A study at the prenatal stage of embryo development showed that terminal breakpoints were an independent predictor of the birth of children with congenital malformations (Shilova et al. 2019).

Previous research suggested the impact of a quadrivalent asymmetry degree on the pattern of chromosome segregation. The results of the study conducted on the cleavage stage embryo demonstrated that severe asymmetry predisposes to the production of genetically unbalanced embryos due to 3:1 segregation (Zhang et al. 2014). At the same time, there were no differences in the frequency of balanced embryos observation at the blastocyst stage; however, ART carriers with severe quadrivalent asymmetry displayed the product of adjacent-2 segregation more often (Zhang et al. 2018).

Several studies emphasized that acrocentric chromosomes involved in rearrangements predispose to 3 : 1 segregation and reduce the incidence of adjacent-1 disjunction (Ye et al. 2012; Yilmaz et al. 2012). In a study conducted on the blastocyst stage, acrocentric chromosomes influenced segregation in combination with strong quadrivalent asymmetry, increasing the frequency of adjacent-2 and 4 : 0 segregation.

The differences in the results of studies conducted on cleavage stage and blastocyst stage embryos can be partly explained by the selection of viable genetically balanced embryos or embryos with relatively small chromosomal imbalances. Therefore, the purpose of this work was to analyze the impact of different factors predisposing to the formation of non-viable embryos with significant chromosomal imbalance due to pathological segregation patterns and influencing the selection of embryos between the third and fifth or sixth days of development.

## ﻿Materials and methods

A retrospective analysis was performed on 39 couples with ART who underwent in vitro fertilization cycles (IVF) with preimplantation genetic testing for structural chromosomal rearrangements (PGT-SR) using fluorescent in situ hybridization (FISH), next generation sequencing (NGS), or array comparative genomic hybridization (aCGH) between 2016 and 2021 at the International Center for Reproductive Medicine. Karyotypes of carriers are presented in Suppl. material 1: table S1. The informed consent form was signed by all the participants. ARTs were confirmed after karyotyping of spouses on peripheral blood lymphocytes. A total of 306 cleavage stage embryos and trophectoderm cells from 93 blastocyst stage embryos were analyzed. The mean age of female ART carriers was 32.3±4 and 34.4±3 in couples who underwent IVF with PGT-SR using FISH or aCGH/NGS methods on the third day and the fifth / sixth day, respectively.

To determine the segregation type in gametogenesis of ART carriers, the combination of fluorescent signals from TS and CS was assessed when analyzing blastomeres of cleavage stage embryos in the case of PGT-SR using the FISH method or by assessing the gain or loss of genetic material when analyzing trophectoderm cells from blastocyst stage embryos in the case of PGT-SR using aCGH or NGS methods.

TS and CS lengths were measured using the UCSC genome browser (assembly GRCh38/hg38) in millions of base pairs to determine the quadrivalent asymmetry degree and the presence of terminal breakpoints. The starting point for measuring the length of the TS was the proximal end of the cytoband. The size of the CS was calculated by subtracting the length of the TS from the length of the entire chromosome.

The quadrivalent asymmetry degree was assessed by calculating the ratio of the length of the longest TS to the shortest TS and the longest CS to the shortest CS. If both ratios were ≥ 2, the quadrivalent was considered severe asymmetric. If at least one of the ratios was less than 2, the quadrivalent was considered mild asymmetric (Zhang et al. 2018).

The ratio of the length of the TS to the length of the entire chromosome arm was measured to determine the presence of terminal breakpoints in ART carriers. A translocation was considered to contain a terminal breakpoint if this ratio was ≤ 0.2 in one or both chromosomes involved in the rearrangement (Ye et al. 2012).

Statistics were calculated using STATISTICA 12 software (Tibco, CA, USA). Fisher’s exact test was used to compare differences between groups.

## ﻿Results

In this article, we analyzed 1) the selection of embryos resulting from different segregation modes by the blastocyst stage; 2) the influence of terminal breakpoints, quadrivalent asymmetry degree, and the involvement of acrocentric chromosomes on the predominant pathological pattern of chromosome segregation in ART carriers; and 3) the influence of the abovementioned factors on the viability of embryos resulting from different segregation patterns.

### ﻿Comparison of the segregation patterns detection frequency in cleavage stage and blastocyst stage embryos

**Figure 1.**
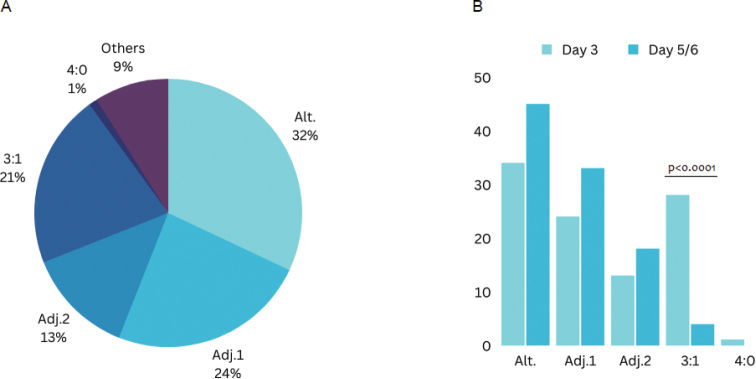
Frequency of observation of different chromosome segregation modes in human embryos **A** from all ART carriers **B** separately in cleavage stage (day 3) and blastocyst stage (day 5/6) embryos. Alt.: alternate mode, Adj.1: Adjacent-1 mode, Adj. 2: adjacent-2 mode.

In total, alternate mode was the most detected segregation pattern and was observed with a frequency of 32% (128/399). Other patterns were detected with comparatively lower frequencies: adjacent-1 and 3 : 1 modes were observed with the same frequency (24% (97/399) and 21% (82/399) respectively); adjacent-2 mode was detected in 13% of embryos (50/399); and 4 : 0 mode was found only in 1% (4/399). The segregation mode was not determined in 9% of embryos (38/399) due to mosaicism or polyploidy, such embryos were excluded from further analysis (Fig. 1A).

When comparing the observation frequency of different types of segregation on days 3 and 5/6 of embryo development, a significantly lower frequency of 3 : 1 segregation was shown in the previous gametogenesis of the carrier (p<0.0001) on day 5/6 embryos, which is explained by the selection of embryos due to the larger size of the chromosomal imbalance, which is typical for this type of segregation (Fig. 1B, Table 1). Segregation modes detected in 3 and 5/6 day embryos are presented in Suppl. material 1: table S2.

**Table 1.** Frequency of segregation modes detection in cleavage stage and blastocyst stage embryos from ART carriers.

Stages of development	Alt., %	Adj.1, %	Adj.2, %	3:1, %	4:0, %
Cleavage	34	24	13	28	1
Blastocyst	45	33	18	4	0
p-value	0.093	0.118	0.281	<0.0001	0.576

Alt.: alternate mode, Adj.1: Adjacent-1 segregation mode, Adj. 2: adjacent-2 segregation mode.

### ﻿Analysis of factors influencing the chromosome segregation pattern in ART carriers

**Figure 2.**
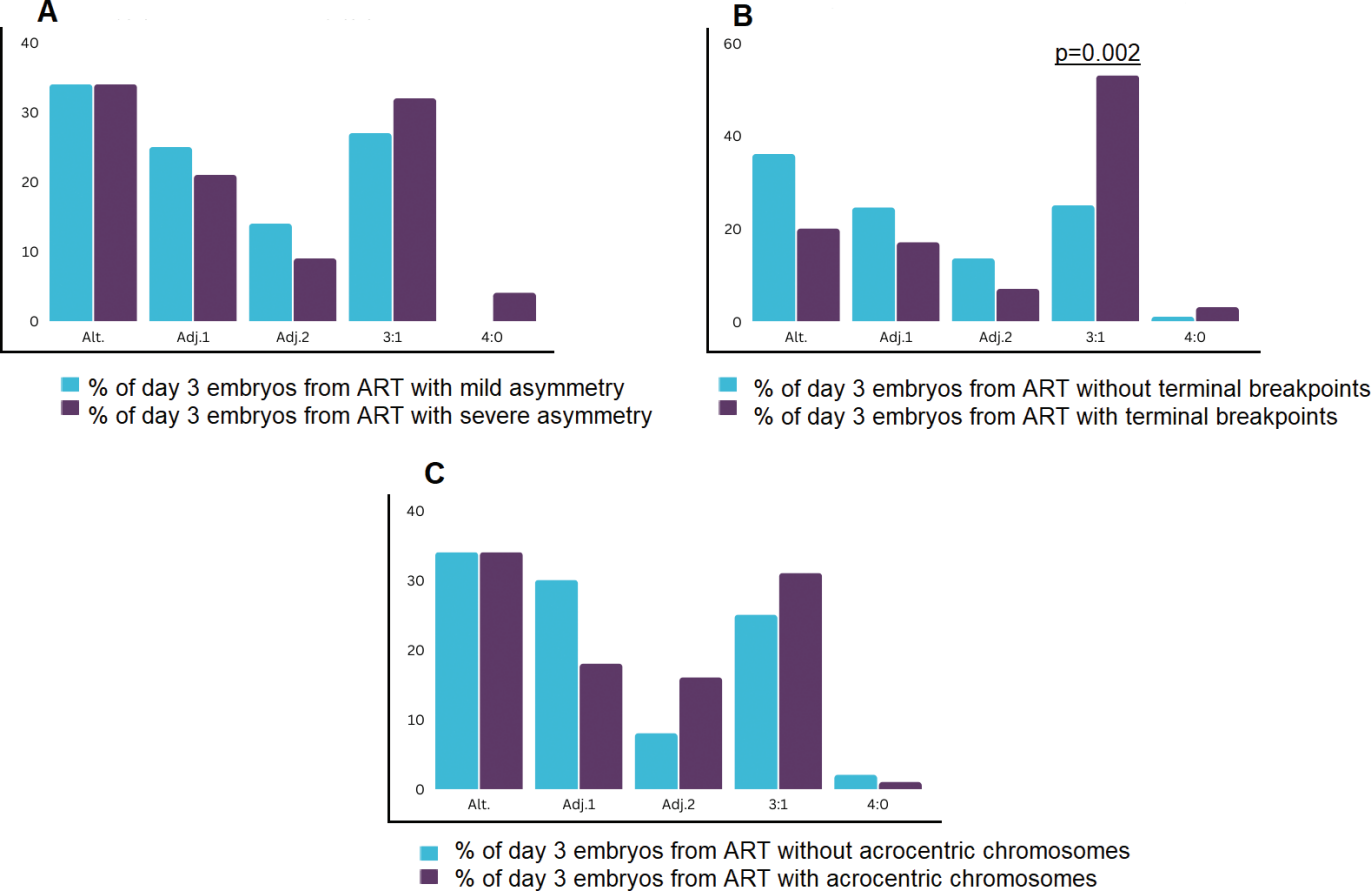
The influence factors on the meiotic chromosome segregation type **A** the quadrivalent asymmetry degree **B** the presence of terminal breakpoints **C** the involvement of acrocentric chromosomes on the meiotic chromosome segregation type. Alt.: alternate segregation mode, Adj.1: Adjacent-1 segregation mode, Adj. 2: adjacent-2 segregation mode.

The direct analysis of gametes is an ideal method for an independent assessment of the influence of factors on the nature of meiotic segregation in ART carriers. Due to the unavailability of gametes for analysis and to exclude the impact of embryo selection by the blastocyst stage the influence of the analyzed factors on the chromosome segregation pattern in ART carriers was assessed on cleavage stage embryos only. Mild quadrivalent asymmetry was determined for 28 ARTs out of 39 (72%). The remaining 11 ART carriers had quadrivalents with severe asymmetry (28%) (Suppl. material 1: tables S1, S3). When assessing the influence of asymmetry degree on the frequency of segregation patterns, no differences were found in cleavage stage embryos (Fig. 2a).

Terminal breakpoints were present in 7 of the 39 translocation (18%) (Suppl. material 1: tables S1, S3). When assessing the influence of the presence of terminal breakpoints on the frequency of segregation patterns, 3 : 1 mode was observed significantly more often in day 3 embryos from ART carriers with terminal breakpoints compared with ART without them (p=0.002) At the same time, genetically balanced embryos were detected with the same frequency (Fig. 2B).

At least one acrocentric chromosome (13, 14, 15, 21, 22) was involved in translocation in 18 of 39 cases (46%) (Suppl. material 1: table S3). When analyzing the frequency of detected segregation patterns in cleavage stage embryos from ART carriers with and without acrocentric chromosomes, it was shown that the involvement of acrocentric chromosomes predisposes to adjacent-2 segregation mode (p=0.046) and reduces the frequency of adjacent-1 mode (p=0,02), without affecting the frequency of balanced embryo formation (Fig. 2C).

### ﻿Analysis of the influence of asymmetry degree, terminal breakpoints, or acrocentric chromosomes’ involvement on the viability of human embryos resulting from different segregation patterns

**Figure 3.**
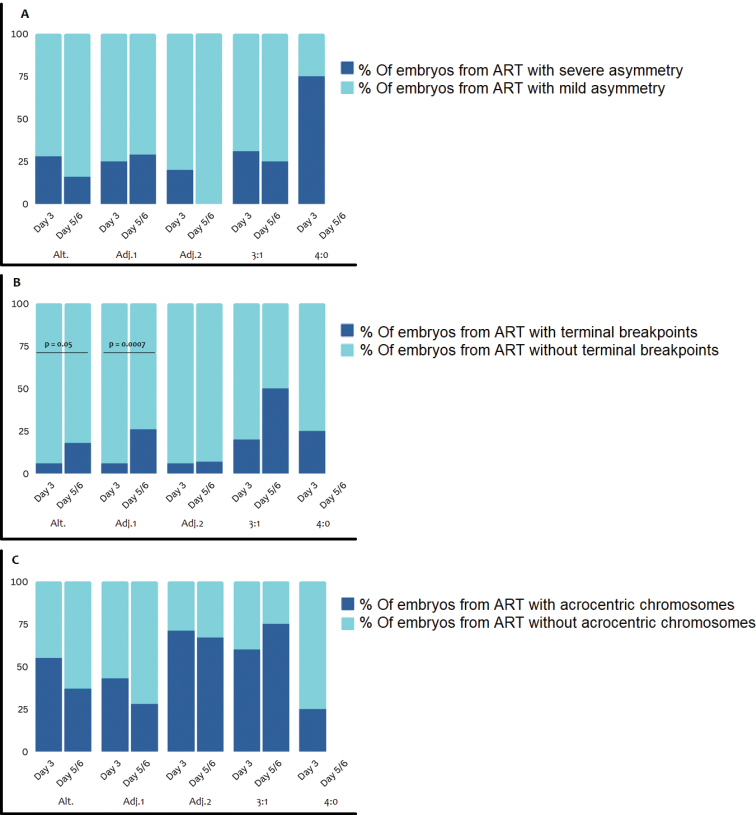
**A** the influence of factors on human embryos survival by the blastocyst stage depending on the type of meiotic chromosome segregation **A** the quadrivalent asymmetry degree **B** the presence of terminal breakpoints **C** the involvement of acrocentric chromosomes. Alt.: alternate segregation mode, Adj.1: Adjacent-1 segregation mode, Adj. 2: adjacent-2 segregation mode.

No statistically significant differences were found regardless of the type of segregation when comparing the frequency of different segregation modes in cleavage stage and blastocyst stage embryos from ART carriers with severe and mild quadrivalent asymmetry, which indicates that quadrivalent asymmetry degree does not affect the viability of human embryos at the initial stages of development (Table 2, Fig. 3A).

**Table 2.** Frequency of segregation modes detection in cleavage stage and blastocyst stage embryos from ART carriers with severe asymmetry degree, presence of terminal breakpoints, or involvement of acrocentric chromosomes.

Types of segregation in ART carriers depending on the presence of factors	Cleavage stage embryos, %	Blastocyst stage embryos, %	p-value
% of embryos with severe quadrivalent asymmetry degree			
Alternate	28	16	0.1861
Adjacent-1	25	29	0.797
Adjacent-2	20	0	0.087
3 : 1	31	25	1
4 : 0	75	0	–
% of embryos with the presence of terminal breakpoints			
Alternate	6	18	0.0513
Adjacent-1	6	26	0.0007
Adjacent-2	6	7	1
3 : 1	20	50	0.208
4 : 0	25	0	–
% of embryos with the involvement of acrocentric chromosomes			
Alternate	55	37	0.0829
Adjacent-1	43	28	0.2474
Adjacent-2	71	67	0.747
3 : 1	60	75	1
4 : 0	25	0	–

When comparing the frequency of segregation modes detected in cleavage stage and blastocyst stage embryos from carriers with terminal breakpoints, a significantly larger number of embryos with alternate (p = 0.05) and adjacent-1 (0.0007) segregation patterns was shown on fifth / sixth days of development.

To assess the effect of acrocentric chromosomes’ involvement on the survival of embryos resulting from different segregation modes, the frequency of detection of different patterns was compared on cleavage stage and blastocyst stage embryos in carriers of ARTs involving acrocentric chromosomes. Such a comparison demonstrated the absence of an impact of acrocentric chromosomes on embryo survival by the blastocyst stage, regardless of the previous segregation pattern (Fig. 3C).

## ﻿Discussion

Our findings on the spectrum of segregation patterns in ART carriers demonstrate the prevalence of an alternate type leading to the formation of gametes forming human genetically normal/balanced embryos (32%). However, the risk of producing genetically unbalanced gametes in carriers remains high, leading to reproductive failure or the birth of offspring with congenital defects (Huang et al. 2019). Moreover, the frequency of observation of pathological segregation patterns is specific to carriers of a particular rearrangement with its individual quadrivalent characteristics. Previous studies suggested factors that potentially affect segregation, such as quadrivalent asymmetry degree, the presence of terminal breakpoints, and the involvement of acrocentric chromosomes. Therefore, in the present study, we analyzed the factors influencing the nature of meiotic chromosome segregation in ART carriers and their effect on the survival of human embryos resulting from different segregation patterns by the blastocyst stage.

Based on the obtained results, the number of human embryos resulting from 3 : 1 segregation in the gametogenesis of ART carriers is significantly reduced by the blastocyst stage, which is most likely due to the significant size of chromosomal imbalance associated with this segregation mode. This finding is consistent with the result of a previous study, which demonstrated a significant decrease in the number of genetically unbalanced embryos resulting from 3 : 1 segregation by the fifth / sixth days of development (Beyer and Willats 2017). At the same time, this study also demonstrated a significant increase in the number of euploid / balanced blastocyst stage embryos resulting from alternate segregation, as well as embryos with small genetic imbalances caused by adjacent-1 mode. In the present study, there is also a minor trend towards an increase in the frequency of detection of alternate and adjacent-1 modes; however, the differences were not statistically significant (0.09 and 0.1, respectively), which can be explained by the relatively smaller sample size (Fig. 1B, Table 1). Selection of embryos formed as a result of adjacent-1 segregation in gametogenesis by the 3^rd^ day of development can be explained by the fact that chromosomal imbalance in this segregation mode is limited to partial monosomy and trisomy of TSs, which are often relatively small compared to CSs. At the same time, the size of the chromosomal imbalance in 3 : 1 segregation is relatively larger compared to adjacent-1 mode, since in this case the imbalance is presented as tertiary or interchange trisomy / monosomy. The early work of Daniel and Cohen allowed the determination of the size of chromosomal imbalance, represented as haploid autosome length (%HAL), potentially compatible with implantation and fetal development. For this, the authors proposed the Chromosome imbalance size-viability Model (Daniel 1979) and Surface of viable unbalances (Cohen 1994). According to the model proposed by Daniel, the size of the viable imbalance does not exceed 2% HAL for monosomy and 4% HAL for trisomy. In Cohen’s modification, these values are 5% and 3% HAL for trisomy and monosomy, respectively. The size of the potentially viable chromosomal imbalance for all the embryos formed as a result of all possible segregation patterns in the analyzed families is presented in Suppl. material 1: table S4. The table shows that the majority of embryos formed after adjacent-1 segregation have a chromosomal imbalance size that is potentially compatible with implantation, which can lead to the birth of a child with congenital anomalies. However, it should be noted that the proposed models for estimating the size of the chromosomal imbalance have not yet been evaluated at the preimplantation stage.

These findings once again confirm the natural selection of human genetically normal/balanced embryos by the blastocyst stage, but at the same time raise the question of factors predisposing to pathological segregation patterns in ART carriers, leading to the formation of genetically unbalanced embryos incompatible with further development. To address the question, the impact of such factors as quadrivalent asymmetry degree, the presence of terminal breakpoints, and the involvement of acrocentric chromosomes on the preferential segregation pattern was analyzed. The assessment was performed on cleavage stage embryos in order to exclude the influence of the natural selection of embryos by the blastocyst stage. According to our results, the only factor analyzed that affects the nature of chromosome segregation in the gametogenesis of ART carriers is the presence of terminal breakpoints predisposing to the 3 : 1 mode. This result confirmed the finding about the incidence of a 3 : 1 pattern in ART carriers with terminal breakpoints, which is significantly higher compared to translocations without them in cleavage stage embryos (Ye et al. 2012).

When comparing the frequency of detection of various types of segregation in cleavage stage and blastocyst stage embryos with severe and mild asymmetry degrees, as well as with and without the involvement of acrocentric chromosomes, no statistically significant differences were revealed regardless of the segregation type, which indicated the absence of influence of these factors on the viability of human embryos at the initial stages of development (Fig. 3A, B).

The opposite trend was demonstrated when comparing the frequency of detection of segregation modes in cleavage stage and blastocyst stage embryos resulting from ARTs with terminal breakpoints and without them. Significantly more embryos consistent with the alternate (p = 0.0513) and adjacent-1 (p = 0.0007) segregation patterns, resulting from translocation with terminal breakpoints, were observed on the fifth / sixth days of development (Fig. 3B). This observation can be explained by the small size of the chromosomal imbalance in the gametes formed as a result of adjacent-1 segregation, which is characterized by partial monosomy or trisomy of TS. The small size of the TS due to the presence of terminal breakpoints leads to the formation of zygotes with a minor chromosomal imbalance, which determines the survival of such embryos by blastocyst stage.

### ﻿Study limitations

A limited number of chromosomes were analyzed in cleavage stage embryos; therefore, selection by blastocyst stage could partly be due to aneuploidy of chromosomes not analyzed. However, selection for chromosomes not involved in the rearrangement should not have affected the frequency of detection of segregation types. The limitations of the study also include the small sample size.

## ﻿Conclusion

Despite the high frequency of alternate segregation in ART carriers, they are at increased risk of reproductive failure or the birth of offspring with congenital defects due to pathological chromosome segregation in gametogenesis. A selection of viable human embryos is observed in the blastocyst stage due to the presence of terminal breakpoints on the chromosomes involved in ART, making PGT-SR of blastocyst stage embryos preferable to the cleavage stage. The presence of terminal breakpoints on the chromosomes involved in the rearrangement promotes the survival of human embryos resulting from adjacent-1 segregation mode with a small size of chromosomal imbalance, increasing the risk of the birth of a child with multiple congenital malformations caused by a chromosomal abnormality.

## ﻿Competing interests

The authors have declared that no competing interests exist.

## ﻿Funding

This research was supported by the Ministry of Science and Higher Education of the Russian Federation (project “Multicenter research bioresource collection “Human Reproductive Health” contract No. 075-15-2021-1058 from 28 September 2021).

## Supplementary materials

Supplementary material 1 Supplementary information Data type xlsx Explanation note **table S1.** Calculation of quadrivalent asymmetry degree and the presence of terminal breakpoints in translocation carriers. **table S2.** Segregation patterns detected in 3- and 5/6 days human embryos. **table S3.** Segregation patterns in 3- and 5/6 days human embryos depending on individual quadrivalent characteristics. **table S4.** Size of chromosomal imbalance and potential viability of all human embryos analyzed. https://binary.pensoft.net/file/971886This dataset is made available under the Open Database License (http://opendatacommons.org/licenses/odbl/1.0/). The Open Database License (ODbL) is a license agreement intended to allow users to freely share, modify, and use this Dataset while maintaining this same freedom for others, provided that the original source and author(s) are credited.Ziravard N. Tonyan, Irina L. Puppo, Alsu F. Saifitdinova, Tatyana V. Vavilova, Andrey S. Glotov
